# Evidence on prevalence of caesarean sections and factors influencing uptake in Ghana: a scoping review protocol

**DOI:** 10.1080/16549716.2025.2563874

**Published:** 2025-10-08

**Authors:** Stella Mahama, Louisa Lawrie, Jamini Dimri, Mairead Black

**Affiliations:** a Ghana Scholarship Secretariat, Accra, Ghana; b Ghana Health Service, Health Promotion Division, Research and Policy Department, Korle Bu, Accra, Ghana; c School of Medicine, Medical Sciences and Nutrition, Institute of Applied Health Sciences, University of Aberdeen, Scotland, UK; d Health Psychology Group, School of Medicine, Medical Sciences and Nutrition, Institute of Applied Health Sciences, University of Aberdeen, Scotland, UK; e Aberdeen Maternity Hospital, Obstetrics Unit, Scotland, UK; f Aberdeen Centre for Women’s Health Research, University of Aberdeen, Scotland, UK

**Keywords:** caesarean sections, caesarean rates, prevalence, factors, optimal use, uptake, barriers, facilitators, Ghana

## Abstract

The World Health Organisation recommends a target rate of 10% to 15% of births by caesarean sections (CS) to save lives. According to the Ghana Demographic and Health Survey report (2022), Ghana’s CS rate is now 21% which is similar to the global rate. The objective of this scoping review is to explore what is known about the prevalence of CS and its uptake across Ghana. Qualitative, quantitative, and mixed-methods studies, as well as published grey literature, exploring the prevalence of CS, as well as the barriers and facilitators influencing CS uptake in Ghana, will be included. This review will be conducted in accordance with the Joanna Briggs Institute methodological guidelines for scoping reviews, and it will report findings in line with the Preferred Reporting Items for Systematic and Meta-Analyses extension for scoping reviews (PRISMA-ScR). Database searches will be conducted in MEDLINE, PsycINFO, EMBASE, Web of Science and CINAHL (EBSCO). The Theoretical Domains Framework will be used to examine the factors influencing healthcare professionals’ recommendations for CS to women, as well as women’s choices regarding undergoing a CS or not. The results will be presented descriptively and in a visual format. The findings will underpin recommendations for future research, policy and practice to support health improvement, and optimal use of CS in Ghana.

## Paper Context

- **Main findings:** We anticipate that the results of this review will include a visual map of Ghana showing the regional variations of the prevalence of CS rates in the country and will outline the barriers and facilitators relevant to specific regions.
- **Added knowledge:** The review intends to map out the published evidence on caesarean section prevalence across Ghana and related barriers and facilitators of its use.
- **Global health impact for policy and action:** The review findings will inform global health policy and practice on the optimal use of CS in Ghana and similar settings.

## Background

The period of pregnancy and the time of birth are critical to the health of pregnant women. The World Health Organisation (WHO) recommends skilled obstetric care for all births in any setting, to optimise health outcomes [1]. Caesarean section (CS), a potentially lifesaving surgical procedure, is recommended to be carried out by highly skilled individuals when medically indicated [2].

Various population-based research studies have revealed that not only is CS a lifesaving procedure, but its utilisation is an indicator of access to skilled obstetric care [1–4] especially in resource-poor settings [5]. However, like any other surgical procedure, CS has the potential for complications in both mother and baby, and in some cases, maternal death [6,7]. It is further associated with both short- and long-term risks. These risks are not exhaustive but include haemorrhage, infection, long recovery times, and complications in future pregnancies [4,8].

In addition to the goal of saving the life of a mother experiencing obstructed labour, CS also aims to deliver a viable baby [9,10]. Common recorded indications for CS include prolonged labour, cord prolapse, foetal distress, abnormal foetal presentation, placental problems, and failed assisted delivery [3,11].

Studies have revealed significant increases in CS rates in both developed and developing countries since the start of this millennia [12]. In this review, developed countries are defined as countries industrialised with high infrastructure, technology, high levels of literacy, quality of life, and Gross National Income (GNI) per capita of 4,516USD and 14005USD [13], while developing countries are those which require more resources, facilities for infrastructure, and are associated with lower levels of literacy and quality of life, with a GNI per capita of up to 1,145USD [13–15]. Several studies have reported that developed countries have been accruing increasing caesarean rates (CR) over time due to high societal acceptance of CS [7]. On the contrary, developing countries have only started to experience an increase in CS owing to minimal acceptance, even when there is a clear danger of aiming for vaginal birth [2,7,9,16]. Furthermore, it is estimated that the rates of CS in developed countries are above the WHO’s recommended range of 10% −15% [17].

Data from 154 countries between 2010 and 2018 revealed that out of 94.5% of the world’s total live births, 21.1% of mothers had a caesarean birth [8]. In their study, Betrán et al. (2007) indicated that globally, the lowest rates of CS are found in Africa (7.3%), and more specifically, 3% in Western Africa, including Ghana [7]. The uppermost caesarean rate within the African continent has been recorded in Northern Africa, with a rate as high as 27.8% [8]. It has been projected that if the 2010 to 2018 trend continues at this pace, then Eastern Asia by the year 2030 will most likely record above 63%, Latin America and the Caribbean 54%, Western Asia 50%, Northern Africa 48% Southern Europe 47% and Australia and New Zealand 45% [18].

The WHO has indicated that the goal of achieving the 10–15% range is to minimise maternal, neonatal, and infant mortality. Above this range, increasing the rate of CS in a given population is not associated with a further decrease in mortality [17].

The International Health Community (IHC), together with the WHO, have acknowledged that one of the considerations that has stalled the understanding of CS trends globally is the absence of a standardised, internationally recognised categorisation process to evaluate CS rates regularly. Both bodies also identified that there are no procedures in place to consistently identify CS trends that cut across different countries, regions, cities, and health facilities [17].

Given that it is unclear why CS rates vary across the world and whether increases in CS rates relate to health benefits (more than just mortality) for mothers and infants, the IHC and the WHO set out to ascertain and understand the gains these elective and emergency surgeries bring to both maternal and newborn outcomes, particularly in developing countries [2,19].

In 2017, the Ghana Health Service (GHS) reported the country’s CS rate as 16% [20]. Given that the WHO recommends a range of 10–15%, Ghana, as of 2017, had joined other countries who have exceeded the WHO target range [17,21]. In the recently released GDHS 2022 report, CS rate in Ghana was 21% of live births [22] which is similar to the global rate.

As suggested by Miller et al., Ghana could have experienced the ’too much, too soon (TMTS)’ phenomenon of obstetric skills delivery [23]. Ghana’s TMTS could have potentially resulted from the over-medicalisation of uncomplicated pregnancies [23]. TMTS involves the excessive use of non-evidence-based interventions that can be lifesaving when used correctly but can be unsafe when used routinely or overused. An increase in the use of facility-based births may have led to this over-medicalisation [22,24].

That aside, there are regional variations in CS rates in Ghana, as well as variations across health facilities [25,26]. For instance, in 2016, the nation’s capital (Greater Accra Region) recorded a CS rate of 24.3% for the region, while rates in the Upper East Region reported rates of 7.2% the same year [20].

Currently, there is no evidence in the literature that proves that the increase in CS rates is an indication of access to quality health care in Ghana [27]. Therefore, this review aims to further understand the extent of variation in CS rate and the underlying reasons for this across Ghana.

Scoping reviews are appropriate for mapping out evidence on the current literature and allowing for the establishment of comprehensive and relevant evidence on a particular field of study. They also allow for the discovery of evidence on relevant concepts and research gaps that inform policy and practice [28]. Considering that scoping reviews are carried out for various purposes, it is important to understand the objective of any scoping review [29]. The study by Peters et al. concluded that scoping reviews are suitable for assessing the scope of evidence in the literature in a particular field of research, or to identify, chart, report, and/or discuss the relevant concepts in a specific research area [30]. This makes the conduct of scoping reviews naturally exploratory [31]. Hence, a scoping review is deemed appropriate to answer the objectives of this proposed study, as it allows the researchers to explore, identify, chart, discuss, and report on published evidence on the prevalence of caesarean sections and factors influencing uptake in Ghana. The review will also explore existing gaps in the literature on the prevalence of CS as well as the barriers and the facilitators influencing its uptake across regions of Ghana and the country as a whole. Furthermore, the review does not intend to conduct a quality assessment of the evidence; thus, conducting a systematic review would not be warranted.

## Methods

This proposed scoping review will be conducted in accordance with the Joanna Briggs Institute methodological guidelines for scoping reviews [32], and it will further adopt and report in line with the Preferred Reporting Items for Systematic and Meta-Analyses extension for scoping reviews (PRISMA-ScR) [33] and its strategy for reporting guidelines and checklist (Appendix S1) to ensure the procedure for synthesising is robust [33–35].

Preliminary searches in JBI Evidence Synthesis (20 October 2023), Prospero (20 October 2023), Cumulative Index to Nursing and Allied Health Literature (CINAHL) (20 October 2023) and Cochrane Database of Systematic Reviews (20 October 2023) did not generate or identify any registered, published, and ongoing systematic or scoping reviews on this research topic.

## Review question

What is known about the evidence on the prevalence of caesarean sections and factors influencing uptake in Ghana?

## Review aim

To identify and synthesise the existing evidence about the prevalence and factors affecting uptake of Caesarean Sections in Ghana.

## Review sub-questions

1. What is the prevalence of caesarean section uptake across Ghana, and how does it vary by region?
2. What are the barriers to CS uptake in Ghana?
3. What are the facilitators of caesarean uptake in Ghana?

## Eligible criteria

### Population

The population of interest is all women who have given birth in Ghana, through CS (either elective or emergency CS) or vaginal birth, in any setting, including home or hospital.

## Inclusion criteria

This scoping review will include primary qualitative, quantitative, and mixed methods studies, as well as systematic and scoping reviews, and published grey literature written in English. Studies or reports will be included if they have explored the prevalence of CS and/or the barriers and/or facilitators influencing the uptake of CS with specific data on Ghana. Studies will be excluded if they do not contain data specific to Ghana.

## Sources for information

Databases searches will include MEDLINE, PsycINFO, EMBASE, Web of Science, and CINAHL (EBSCO). Grey literature will be identified from Google Scholar and Google websites that are noted to publish literature on CS, including the WHO, World Bank, Lancet, Ghana Health Service, UNICEF, AMDD, UNFPA, and the International Journal of Gynaecology and Obstetrics. The research team will source prevalence data on CS from websites including the African Index Medicus, INDEPTH Network, DHS program, Ghana Statistical Service, Ghana Health Service, Ghana Demographic Health program, Ghana Health and Demographic Surveillance System, Ghana Emergency and Obstetric and Newborn Care, ICF International, World Bank, and other websites of organisations that report on CS in Ghana.

## Search strategy

In accordance with the JBI guidance, the search strategy will follow three steps [33]. The first step will be the first limited search that will be conducted in Ovid MEDLINE, PsycINFO, EMBASE, Web of Science, and CINAHL (EBSCO). This step will be carried out to retrieve eligible papers pertaining to the research topic and the objectives of the review. As part of this initial stage, text words and index terms used to describe the relevant papers will be identified and subsequently used to develop a broad search strategy to retrieve relevant papers to address the objectives of the review. Table 1 illustrates the planned initial search strategy, including the keywords, synonyms, Boolean operators, and Medical Subject Headings (MeSH) terms, to be conducted in Ovid Medline (step 1). In step two, the full second search will be conducted in the five databases: MEDLINE, PsycINFO, Embase, Web of Science, and CINAHL. In step three, further searches will be conducted of the reference list of all papers included in this review.

**Table 1.** Full search strategy on Ovid Medline.

Number	Search Terms	Results
1	’‘caesarean section*’’.mp.	21487
2	’‘cesarean section*’’.mp.	68255
3	’‘c-section*’’.mp.	2102
4	1 or 2 or 3	78284
5	prevalence*.mp.	913617
6	rate*.mp.	3821880
7	epidemiology*.mp.	2279076
8	uptake*.mp.	463936
9	barrier*.mp.	437323
10	facilitator*.mp.	41054
11	factor*.mp.	6713414
12	determinant*.mp.	299344
13	15 or 6 or 7 or 8 or 9 or 10 or 11 or 12	11367894
14	4 and 13	37652
15	Ghana*.mp.	17854
16	West Africa*.mp.	15486
17	15 or 16	31995
18	14 and 17	151
19	Limit 18 to (English language and humans)	123

Furthermore, relevant journals, including the Pan African Medical Journal, Journal of Public Health in Africa, International Journal of Gynaecology and Obstetrics, and the Ghana Medical Journal, will be searched to collect relevant published information regarding CS in Ghana.

In addition, an author search will be conducted on journals, whereby the names of authors of relevant articles will be searched to retrieve additional and relevant articles published by them [36]. No date restrictions will be applied to this review. Eligible papers will be restricted to those published in English.

## Study and source of evidence selection

**Figure 1.**
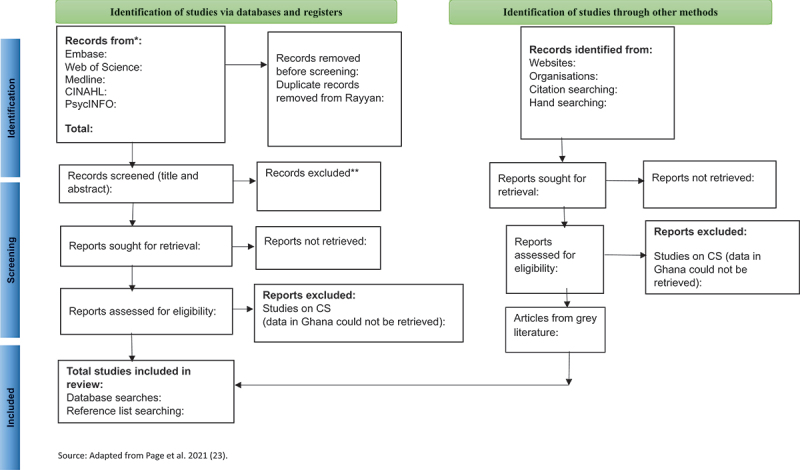
Template PRISMA flow diagram for database searches and other sources.

The pool of search results from the five databases will be exported into Refworks, a referencing manager. Duplicated articles will be removed. The remaining articles will then be screened by title and abstract to identify those that potentially meet the inclusion criteria. The Rayyan web tool for screening articles will be used by the reviewers to double-screen titles and abstracts [37]. The potentially relevant sources of evidence will then be screened in full text. This stage will be an independent exercise carried out by a three-member review team made up of the primary researcher (SM), a second reviewer (JD), and the project supervisor, MB (third reviewer), who will act as arbitrator for any articles where agreement has not been reached by the first and second reviewer. The results of the search will be presented and reported in the form of a PRISMA-ScR flow chart diagram template [38] (Figure 1). The reasons for excluding studies will be provided in the review.

## Data extraction

The data extraction template from the JBI Evidence Synthesis manual [33] will be adapted to (Appendix S2) identify and chart the data pertaining to the prevalence of CS, the barriers, and facilitators to its uptake in Ghana. The data extraction form will collect the following details: study, name of first author and year, study country/region, aim(s) of the study, study method/design, type of data collection/approach, population size (number of women), prevalence rates of CS per study region, key findings on CS barriers by the primary author(s) and key findings on CS facilitators by the primary author(s).

In addition, the data extraction for this review will employ the use of both inductive and deductive (qualitative) methodologies. The application of the inductive qualitative analysis is valuable for developing themes that are not associated with an existing theoretical framework, given the exploratory nature of the topic. The deductive analysis will be advantageous for delineating themes that are predetermined or anticipated, based on the Theoretical Domains Framework (TDF) [39].

At least 20% of the data will be checked for accuracy by a second reviewer. To ensure that the data extraction template will be optimal for use, the reviewers will independently test the data extraction template on three randomly selected papers [33,40].

## Data analysis, interpretation, and presentation

The prevalence of CS in Ghana will be analysed using data from each included study. Graphs and maps will be used to provide an illustration of the most recent prevalence rates of CS across Ghana. The TDF will be used to facilitate the analysis of barriers and uptake of CS across Ghana. TDF is a framework used to categorise and explain why a certain behaviour occurs and/or used to determine the factors influencing behaviour among health professionals and patients.

The TDF framework is appropriate in this review because we aim to explore and identify the influencers of the behaviour of healthcare professionals and those of patients (women) on the decisions based on the uptake of CS [39]. Notably, the TDF is a useful framework as it integrates multiple theories of behaviour into a single framework that is accessible to use during analysis [41].

In this review, factors influencing healthcare professionals’ recommendations for CS to women, as well as women’s choices regarding undergoing a CS or not, will be examined in the context of the TDF [39].

In addition, the TDF will be used as a theoretical basis to explore the behavioural factors influencing the barriers and facilitators to the uptake of CS, structure the evidence synthesis, and show a graphical depiction of the findings on the barriers and facilitators to uptake of CS in Ghana [39]. The data on barriers and facilitators will be presented in a table format and were considered helpful; further representation will be developed in the form of hierarchical graphs to display the most relevant/frequent domains. Furthermore, a comprehensive narrative summary of the data extracted will be conducted [33]. Finally, the evidence gathered and the gaps in knowledge identified in this research topic will be used to draw up recommendations for further studies and to inform health improvements on the uptake and optimal use of CS in Ghana.

## Handling conflicting findings

The review will present and discuss any conflicting evidence (e.g. differences in perspectives from HCPs and mothers)

## Ethical approval

The review’s protocol will use existing published data; thus, no ethical challenges are anticipated. We acknowledge that there may be ethical issues within the existing primary studies that underpin this work. However, ethical approval is not required for this scoping review.

## Discussion

This scoping review will provide a detailed understanding of how CS rates vary across Ghana and the likely underlying reasons for this variation. The review is likely to highlight gaps in knowledge on the barriers and the facilitators of CS uptake in specific regions of Ghana, which will direct future research.

The scoping review will describe examples of how each TDF domain may affect the uptake of CS in Ghana. For example, studies that describe how social support impacts the decision to undergo a CS would typically fall under the ‘social influences’ TDF domain. Identifying the relevant domains that affect CS uptake will be necessary to inform interdisciplinary efforts to optimise the use of CS in Ghana.

To the best of the author’s knowledge, no review has explored the prevalence of CS and the factors influencing the uptake in Ghana to date. As such, this scoping review intends to inform future research on this topic.

The limitation in this scoping review is that studies not published in English will be excluded.

## Supplementary Material

GHA_Appendices_1_and_2_org.docx

## Data Availability

No datasets were generated or analysed during the current study. All relevant data from this study will be made available upon study completion.
